# Adherence to the Mediterranean Diet among Children and Youth in the Mediterranean Region in Croatia: A Comparative Study

**DOI:** 10.3390/nu14020302

**Published:** 2022-01-12

**Authors:** Antonela Matana, Ivana Franić, Endica Radić Hozo, Ante Burger, Petra Boljat

**Affiliations:** 1 The University Department of Health Studies, University of Split, Ruđera Boškovića 35, 21000 Split, Croatia; ivana.zorica@ozs.unist.hr (I.F.); erhozo@ozs.unist.hr (E.R.H.); anteburger@gmail.com (A.B.); 2 Elementary School Žnjan-Pazdigrad, Pazdigradska 1, 21000 Split, Croatia; petraa.boljat@gmail.com

**Keywords:** Mediterranean diet, children, youth, kindergarten, primary school, secondary school, faculty

## Abstract

The Mediterranean diet (MD) is considered one of the healthiest dietary patterns. The aim of this study was to assess MD adherence in children and youth living in the Mediterranean region in Croatia and evaluate the differences in adherence to the MD among different educational stages. In total, 2722 individuals aged 2 to 24 years were enrolled in this study. Subjects were divided into different groups according to the Croatian educational system. Mediterranean Diet Quality Index (KIDMED) was used to assess adherence to the MD. In the total sample, the adherence to the MD was poor in 19.2%, average in 60.8%, and good in 20.1% of the study participants. The prevalence rate of poor adherence to the MD increased with higher educational stage, i.e., the highest prevalence rate of poor MD adherence was observed for college students (39.3%). Children having a higher number of snacks on days-off, those with lower physical activity, and not having breakfast together with a family are more likely to have poor MD adherence, while children having a higher number of snacks on working days are less likely to have a poor MD. The results of this study showed low adherence to the principles of the MD, confirming the need for improvement of adherence to the MD pattern in the studied population.

## 1. Introduction

The Mediterranean diet (MD) is considered one of the healthiest dietary patterns in the world, characterized by high consumption of fruits, vegetables, legumes, olive oil, nuts, and cereals, a moderate-high intake of fish, dairy products, and alcohol (mostly wine), and a low intake of saturated lipids, sweets, and red and processed meat [1,2]. Numerous studies have shown that adherence to the MD is associated with a significant reduction in total mortality and improvement in longevity, as well as with various health benefits, including prevention of cardiovascular diseases, neurodegenerative diseases, diabetes type 2, obesity, cancer, and many others [3,4,5,6,7,8].

However, despite all the existing evidence about the benefits of this diet, a transition from this dietary pattern towards a high-energy diet style, which is rich in saturated fats and low in micronutrients, has been observed, especially in the younger generation [9]. This change has led to an increase in obesity and numerous negative health-related consequences [6,10]. Childhood obesity is a particular public health concern [11]. The results of the European Childhood Obesity Surveillance Initiative in Croatia in 2018/2019 indicate that 35% of children aged 8.0 to 8.9 years had overweight or obesity [12]. In addition, according to the research conducted by the Organisation for Economic Cooperation and Development (OECD), in the next 30 years, life expectancy in Croatia will be shortened by 3.5 years due to overweight [13].

Healthy lifestyle habits develop in the early stages of life and impact human health significantly in later life, making childhood and adolescence particularly important for the adoption and maintenance of healthy habits [14]. So far, only a few studies on adherence to the MD among the Croatian young population have been performed [15,16,17]. A study recently conducted among Croatian university students showed that college students have poor eating habits, with 42.8% of students having low MD adherence scores [15]. Surprisingly, given the fact that kindergartens in Croatia follow institutionalized nutritional recommendations and that children spend most of their daytime hours in kindergartens, results for preschoolers in Croatia are inconsistent: while one study showed that only 6% of the children had a low MD adherence score (12), other recently published research revealed that almost half of the study participants (49%) had a low KIDMED index score [17].

To the best of our knowledge, there has been no study performed specifically among primary and secondary school children in Croatia, thus the question of what happens to children‘s eating habits after kindergarten age, especially considering that most of Croatian primary and secondary schools do not provide institutionalized feeding for their students, remains unanswered. Results of published studies suggest that good eating habits are lost by the time of university study, so it is necessary to determine at what age children’s habits begin to change so that additional efforts can be made on time to educate children about the benefits of proper nutrition.

Given the aforesaid, estimating MD adherence and exploring potential predictors might be useful for developing strategies for improving diet quality. Therefore, the main aim of this study was to assess MD adherence in the youth population living in the Mediterranean region in Croatia and evaluate the differences in adherence to the MD and its components among preschool, primary, secondary school children and students by using the same validated questionnaire KIDMED for all age groups [18].

## 2. Materials and Methods

### 2.1. Study Design

This cross-sectional study was carried out from September to November 2021 in children and youths from the Mediterranean region of Croatia (including the regions of Istria, Kvarner, Dalmatia, the Dubrovnik area, and the Adriatic Islands). Participants were aged from 2 to 24 years and were enrolled in randomly selected public kindergartens, elementary or secondary schools, or faculties. The final sample comprised 2722 eligible participants. Participants were categorized into 5 groups according to the Croatian educational system: (i) kindergartens, (ii) primary schools (1st–4th grade), (iii) primary schools (5th–8th grade), (iv) secondary schools, and (v) faculties (college students). The study was approved by the Ethics Committee of the University Department of Health Studies, University of Split (Class 001-01/21-01/01, reg. no.: 2181-228-103/1-21-22) and was conducted in regulation with the latest Helsinki declaration. The subjects gave consent to participate by submitting a completed questionnaire.

### 2.2. Questionnaire

Data were collected using the anonymous questionnaire. Based on the study site preferences, the questionnaire was delivered either as a paper-based or online survey. The online survey was constructed with the Google Forms application and was distributed among study sites using email. The expected time to complete the survey was 10 min. For the purpose of this study, the questionnaire was completed by the child’s parent for participants enrolled in kindergartens, primary and secondary schools, while university students filled out a questionnaire on their own. Only one child per household was included in the study.

The questionnaire consisted of four sections. In the first section, we collected general information about participants, including gender, age, type of study program, year of attendance and parent-reported (or self-reported for students) weight (in kg) and height (in cm). Additionally, the participant’s general health (self- or parent-perceived) was rated as excellent, very good, good, or fair/poor. Body mass index (BMI) was calculated as weight divided by height squared (kg/m^2^), in order to calculate the BMI-for-age percentiles using the CDC growth charts. According to the CDC classification, percentiles lower than 5th is considered as underweight, percentiles between 5th and 85th are considered as normal weight, percentiles between 85th and 95th are considered as overweight, and percentiles ≥95th are considered as obese. Specifically, according to the World Health Organization standards, students were considered to be underweight if their BMI was lower than 18.5, normal weight if the BMI was 18.5 to 24.9, overweight if the BMI was 25 to 29.9, and obese if it was greater than 30 [19].

The second section consisted of two questions regarding physical activity: “Do you/Does your child participate in organized physical activity (possible answers: Yes/No)?”, “How many times a week do you (or does your child) do some sport, dance, or play a game in which you are (or your child is) very active? (possible answers: none, 1 time, 2–3 times, 4–5 times, 6 or more times)”.

Dietary habits, such as the number of main meals and snacks (recorded separately for working days and off-days), as well as information on eating breakfast, lunch, and dinner together as a family, were assessed in the third section.

In the last section, the level of adherence to the MD for participants was evaluated using the KIDMED test (Mediterranean Diet Quality Index for children and adolescents) [18]. KIDMED is a questionnaire consisting of 16 yes or no questions. Questions with a negative connotation with a respect to MD were given a score of -1 (including consumption of fast food, baked goods, sweets, and skipping breakfast), and those with a positive connotation were given a score of +1 (consumption of oil, fish, fruits, vegetables, cereals, nuts, pulses, pasta or rice, dairy products, and yogurt). The total score ranges between −4 to 12 and is classified into 3 levels: (i) low MD adherence: KIDMED score ≤3; (ii) average MD adherence: KIDMED score 4–7; (iii) good MD adherence: KIDMED score ≥8. The instrument was originally developed to assess the level of adherence to the MD in Spanish children and adolescents aged 2 to 24, and was previously adapted for the Croatian language and tested for reliability and validity [15].

### 2.3. Statistical Analysis

The Kolmogorov-Smirnov test was used for normality checking. Due to the non-normal distribution of the data, continuous variables are presented as the median (interquartile range, IQR). Categorical variables are presented with frequencies (percentages). Differences in categorical variables were analyzed by using a Chi-square test, while the Kruskal-Wallis test was used for not normally distributed continuous variables.

Furthermore, we performed multivariate multinomial logistic regression in order to assess the association of MD adherence categories with the odds ratios of predictors that were significant in univariate models (including age, number of daily meals and snacks both on working days and days-off, two questions regarding physical activity, and having breakfast and dinner together as a family). BMI categories and having lunch together as a family were not significantly associated with MD adherence in the univariate model, therefore were not included in the final model.

Finally, a multivariable multinomial logistic regression was also employed to assess the simultaneous effect of MD adherence and level of physical activity on self-perceived health. *p*-values of less than 0.05 were considered statistically significant. Statistical analysis was conducted using Statistical Package Software for Social Science, version 28 (SPSS Inc., Chicago, IL, USA).

## 3. Results

A total of 2722 children and youths participated in this study. Basic characteristics of the study participants are presented in Table 1.

In the total sample, the median KIDMED index score was 6 (IQR: 3), while the adherence to the MD was poor in 19.2%, average in 60.7%, and good in 20.1% of the study participants. The highest compliance to the KIDMED items was observed for eating fast food less than once a week, the consumption of olive oil at home, eating breakfast, and the consumption of dairy products for breakfast (Table 2). No significant gender differences were observed for MD adherence categories (*p* = 0.146). Age was significantly associated with MD adherence categories (participants with poor MD adherence were the oldest, followed by the average group, while the youngest were participants from the good MD category (*p* < 0.001)). Although a higher prevalence of poor MD adherence was recorded among obese (25.6%) and overweight (20.8%) individuals compared to those with normal BMI (18.6%) or underweight (16.5%), no statistically significant association was observed for BMI categories and MD adherence (*p* = 0.120).

**Figure 1:**
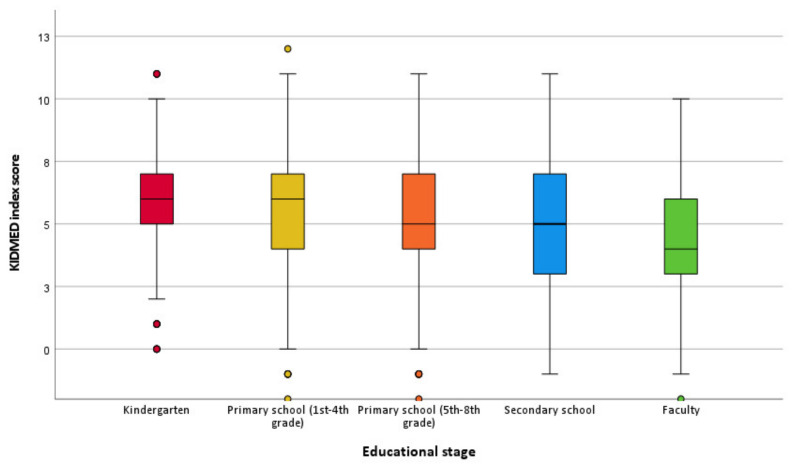
Box–plots for the KIDMED index score for different educational stages.

Regarding the differences in adherence to the MD for different educational stages, the results showed that the KIDMED index score decreased with higher educational stage, i.e., the highest KIDMED index score was observed for children enrolled in kindergartens, followed by children from the first four grades of primary schools, then children from grades 5–8 of primary schools and youths enrolled in secondary schools, while the lowest score was observed for students (*p* < 0.001) (Figure 1). The prevalence rate of poor adherence to MD also increased with higher educational stage (Table 2). The highest prevalence rate of poor MD adherence was observed for students (39.3%), then for children from secondary schools (25.7%), followed by primary school children (16.8% for 1st–4th grades and 19.6% for 5th–8th grades), and the lowest rate was observed for the children enrolled in kindergartens (11.3%) (*p* < 0.001) (Table 2).

Basic descriptive statistics of the 16 KIDMED items according to educational stage is presented in Table 2. The prevalence rate of daily consumption of fruit or fruit juice, a second serving of fruit, fresh or cooked vegetables, as well as fish consumption more than 2–3 times a week, consumption of olive oil and dairy products for breakfast decreased with higher educational stage, while the prevalence rate of regular nut consumption and consumption of pasta and rice increased with higher educational stage. Children enrolled in kindergarten or first four grades of elementary school less frequently ate fast-foods and commercially baked goods or pastries for breakfast compared to higher educational stages. Students consumed cereals or cereal products for breakfast less frequently compared to individuals in other educational stages. The prevalence of skipping breakfast was higher for higher educational stages (Table 2).

**Figure 2:**
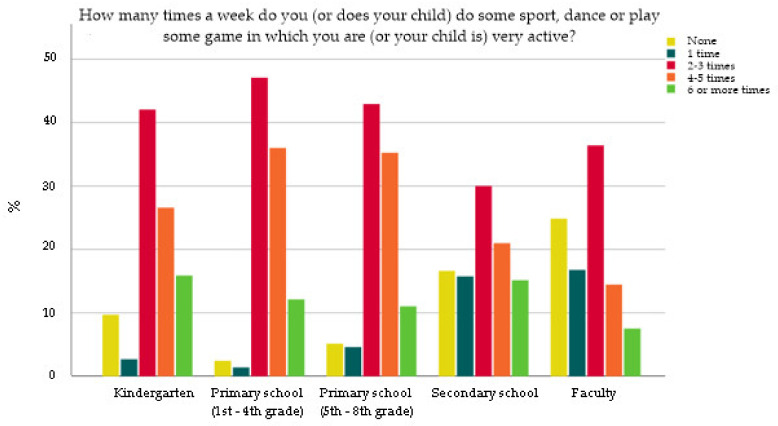
Distribution of answers to the question “How many times a week do you (or does your child) do some sport, dance or play some game in which you are (or your child is) very active?”.

Regarding physical activity, on the question “Do you/Does your child participate in organized physical activity?” the highest percentage of elementary school participants (78.7% for 1st–4th grades and 75.4% for 5th–8th grades, respectively) answered affirmatively compared to participants from high schools (47.5%), kindergartens (40.2%) and faculties (30.1%) (*p* < 0.001). Distribution of answers to the question “How many times a week do you (or does your child) do some sport, dance or play a game in which you are (or your child is) very active?” is shown in Figure 2. The “None” answer was more frequently chosen by students compared to other educational groups (Figure 2) (*p* < 0.001).

Furthermore, we performed a multivariate multinomial logistic regression for MD adherence with nine predictors listed in Table 3 (*p* < 0.001, Nagelkerke R2 = 0.103, correct prediction rate 61.9%) which were significant in the univariate model. The results of the multivariate model are presented in Table 3. Children having a higher number of snacks on days-off, those with lower physical activity, as assessed with the question: “How many times a week do you (or does your child) do some sport, dance or play some game in which you are (or your child is) very active?” and not having breakfast together with a family are more likely to have poor MD adherence, while children having a higher number of snacks on working days are less likely to have a poor MD adherence than average and good MD adherence (Table 3). Additionally, older participants were less likely to have average MD adherence than poor MD adherence.

The two-predictor model of participant’s general health (*p* < 0.001) showed that both, lower level of physical activity and poor MD adherence, were associated with worse self-assessment of health, accounting for 6.3% of the total variance (Nagelkerke R^2), and the correct prediction rate was 58.8% (Table 4).

## 4. Discussion

The main purpose of this study was to evaluate the differences in adherence to the Mediterranean diet and its components according to different educational stages in children and youths living in the Mediterranean region in Croatia. To the best of our knowledge, this is the first study that evaluates and compares MD adherence in individuals from all educational stages, including kindergartens, primary and secondary schools, and faculties.

Our results demonstrated a rather low prevalence of good adherence to the MD over the entire sample (20.1%). This result is in line with a study performed in the Croatian adult population, in which only 23% of the participants from Southern Dalmatia adhered to the principles of MD [20]. Furthermore, we showed that the prevalence rate of poor adherence to the MD increased with higher education stage. The highest prevalence rate of poor MD adherence was recorded among students (39.3%), then for children from secondary schools (25.7%), followed by primary school children (19.6% for 1st–4th grades and 16.8% for 5th–8th grades), while the lowest rate was observed for the children enrolled in kindergartens (11.3%). These results were somehow expected, given the fact that kindergartens in Croatia follow institutionalized nutritional recommendations which promote increased consumption of vegetables, fruits, meat, fish, and dairy products, while the great majority of primary and high schools do not provide institutionalized feeding for their students [16]. Besides the probable influence of institutionalized feeding, this result could also be explained by parental supervision and control over children’s diets, an influence that is gradually lost as children grow up. Several studies have shown that parental control is associated with following healthy dietary habits [21,22,23]. Additionally, another potential factor associated with food choice is pocket money amount which usually increases with age, and increases the probability of consumption of unhealthy fast-food, baked goods, and sweets [24]. Moreover, it is known that students are more likely to buy foods that are fast, convenient, and inexpensive [25]. In the study performed by Marquis et al., it was also shown that college students often make food choices based on cost and convenience over health [26]. Our finding that the prevalence rate of poor adherence to the MD increased with higher education stage is in line with other similar studies performed in Spain and Italy [18,24,27,28]. Croatian data on children and young individuals are relatively few and mainly refer to preschool children and students. We performed the largest study of MD adherence in the Croatian youth population so far by including 2722 subjects. Our results for students are in accordance with the study from Štefan et al., in which 42.8% of college students had poor compliance with MD [15]. However, as already mentioned, results for the preschool population are contradictory, while the results of one study showed that only 6% of children had low KIDMED score [16], the other identifier of significantly higher prevalence (49%) of low adherence to the MD [17]. In both studies, only children from the urban area of Split-Dalmatia County were examined, while our study included subjects from the entire Mediterranean region of Croatia. To the best of our knowledge, there is no published study performed among primary and secondary school children in Croatia, so the results of this study are of particular interest for this young population.

A statistically significant difference in the KIDMED score with regard to sex and BMI categories was not found in our study, which is in line with the results of the majority of other studies, including the systematic reviews of European data [17,29,30,31,32].

Moreover, the multinomial logistic regression results identified several predictors of MD adherence. Children not having breakfast together with a family, having a higher number of snacks on days-off and a lower number of snacks on working days, and those with lower physical activity are more likely to have poor MD adherence.

Several studies have shown that eating at least one meal per day with a family member has a positive impact on general health and avoiding obesity [33]. As in the present study, studies carried out in Italy and Spain also observed a significant association between MD adherence and eating breakfast with the family [34,35].

Interestingly, our results indicate that children having a higher number of snacks on days-off and a lower number of snacks on working days are more likely to have poor MD adherence. Actually, several studies conducted in the Nordic countries and in the United States have observed differences in dietary quality on working days compared to the weekend [36,37,38,39,40,41]. The study in Swedish children has shown that children had their highest intake of sucrose on Fridays and Saturdays due to increased intake of sweets and soft drinks [38]. Another study also confirmed that the intake of total sugars and foods and drinks rich in added sugar were generally higher on weekends versus weekdays for children in Hungary, Italy, and Sweden [36]. Furthermore, during days off, children have more free time and are likely to spend more time in front of screens (watching television, using computers, tablets, and smartphones, and playing video games) [42]. Screen time is usually associated with sedentary behavior and snacking which is often characterized by low nutritional quality [43]. Moreover, another study has shown that a greater amount of screen time is associated with lower consumption of healthy food, including vegetables, legumes, fish, and nuts, and greater consumption of sweets and fast food which consequently leads to poor adherence to the principles of MD [44].

Regarding physical activity, our results are in accordance with previous reports, showing a positive association between physical activity and other healthy lifestyle habits including proper nutrition and MD adherence [24,31,45,46,47,48,49]. A possible explanation for this association is that those children who are physically active and eat healthily are probably adequately educated and coached by their parents. Indeed, previous reports of a positive association between general parental support and physical activity among youth have been recorded [50,51,52]. Results from an above mentioned study performed in Croatian preschoolers also confirmed a positive association of physical activity and KIDMED scores [16], while other Croatian studies performed in youths did not examine physical activity [15,17]. Furthermore, the results of the present study confirmed that a combination of adherence to MD and high physical activity is beneficial to participants’ parent- or self-rated health, and underscore the importance of adopting healthy lifestyle habits for better general health. Although overall health status in the present study was self- or parent-reported, it has been shown that self-perceived health is a valid proxy indicator of health status [53] and that both child and parent reports for health-related quality of life are valid [54].

Our study has several limitations that need to be mentioned. The main limitation is the cross-sectional design, which limits inference on causality. Second, the parents filled out the questionnaire (for all study subjects except for the students), so there is a possibility of parental overestimation or underestimation of the quality of a child’s nutrition. Data on some other potential predictors of adherence to an MD were not collected, such as sleep habits and socio-economic data. On the other hand, this is the most comprehensive study conducted so far on MD adherence in the youth population in Croatia, the results of which have undoubtedly improved existing knowledge on adherence to an MD among children and youth in the Mediterranean region of Croatia. We have included subjects from a larger geographical region and within a wider age range compared to previously published studies in preschool children and students. Even more, to the best of our knowledge, we performed the first study on primary and secondary school children from Croatia and provided crucial data on dietary habits for this population. Another strength of this study is that we used the same validated questionnaire KIDMED for all educational stages, including university students, which enables us to directly compare the results among groups.

## 5. Conclusions

To conclude, the results of this study showed low adherence to the principles of the MD, confirming the need for the improvement of adherence to the MD pattern in the studied population. The present study provided crucial information on MD adherence in different educational stages and helped in defining periods when dietary habits are less healthy. This can help in developing tailored nutritional programs since it was shown that strategies designed explicitly to subgroups are needed. The findings of this study underscore the need to advise and motivate the young population so that healthy dietary habits can be integrated into their lifestyle.

## Figures and Tables

**Table 1:** Basic characteristics of the study participants.

Variable	Descriptive Statistics
Gender, *n* (%)	
Females	1340 (49.2%)
Males	1382 (50.8%)
Age, median (interquartile range)	10.0 (6.0)
BMI classification, *n* (%)	
Underweight	199 (7.3%)
Normal weight	1884 (69.2%)
Overweight	351 (12.9%)
Obese	163 (6%)
Educational stage, *n* (%)	
Kindergarten	485 (17.8%)
Primary school (1st–4th grade)	941 (34.5%)
Primary school (5th–8th grade)	780 (28.6%)
Secondary school	343 (12.6%)
Faculty (college students)	173 (6.3%)

**Table 2:** Results of the KIDMED test according to educational stage.

	Total Sample	Kindergarten	Primary School (1st–4th Grade)	Primary School (5th–8th Grade)	Secondary School	Faculty	*p* Value
KIDMED index score, *n* (%) ^1^							
Poor	523 (19.2%)	55 (11.3%)	158 (16.8%)	153 (19.6%)	88 (25.7%)	69 (39.9%)	<0.001
Average	1653 (60.7%)	315 (64.9%)	599 (63.7%)	462 (59.2%)	196 (57.1%)	81 (46.8%)	
Good	546 (20.1%)	115 (23.7%)	184 (19.6%)	165 (21.2%)	59 (17.2%)	23 (13.3%)	
KIDMED items, *n* (%) ^2^							
Fruit or fruit juice daily	2321 (85.3%)	455 (93.8%)	800 (85%)	652 (83.6%)	280 (81.6%)	129 (74.6%)	<0.001
Second serving of fruit daily	1271 (46.7%)	260 (53.6%)	437 (46.4%)	369 (47.3%)	144 (42.0%)	60 (34.7%)	<0.001
Fresh or cooked vegetables daily	1942 (71.4%)	376 (77.5%)	681 (72.4%)	538 (69%)	227 (66.2%)	117 (67.6%)	0.003
Fresh or cooked vegetables > 1/day	585 (21.5%)	107 (22.1%)	199 (21.1%)	172 (22.1%)	70 (20.4%)	37 (21.4%)	0.749
Regular fish consumption (at least 2–3/week)	658 (24.3%)	142 (29.3%)	230 (24.4%)	177 (22.7%)	77 (22.4%)	28 (16.2%)	0.010
	**Total Sample**	**Kindergarten**	**Primary School (1st–4th Grade)**	**Primary School (5th–8th Grade)**	**Secondary School**	**Faculty**	***p* Value**
>1/week fast-food (hamburger) restaurant	130 (4.8%)	9 (1.9%)	17 (1.8%)	32 (4.1%)	41 (12%)	31 (17.9%)	<0.001
Pulses > 1/week	1556 (57.2%)	263 (54.2%)	545 (57.9%)	461 (59.1%)	194 (56.6%)	90 (52%)	0.429
Pasta or rice almost daily (≥5 days/week)	427 (15.7%)	49 (10.1%)	125 (13.3%)	113 (14.5%)	76 (22.2%)	62 (35.8%)	<0.001
Cereal or cereal product for breakfast	1555 (57.1%)	274 (56.5%)	559 (59.4%)	459 (58.8%)	200 (58.3%)	60 (34.7%)	<0.001
Regular nut consumption (at least 2–3/week)	1054 (38.7%)	172 (35.5%)	346 (36.8%)	305 (39.1%)	148 (43.1%)	81 (46.8%)	0.041
Use of olive oil at home	2486 (91.4%)	463 (95.5%)	862 (91.6%)	701 (89.9%)	306 (89.2%)	149 (86.1%)	0.003
No breakfast	299 (10.99%)	17 (3.5%)	45 (4.8%)	96 (12.3%)	73 (21.3%)	67 (38.7%)	<0.001
Dairy product for breakfast	2413 (88.7%)	446 (92%)	856 (91%)	700 (89.7%)	291 (84.8%)	116 (67.1%)	<0.001
Commercially baked goods or pastries for breakfast	1173 (43.1%)	167 (34.4%)	383 (40.7%)	361 (46.3%)	176 (51.3%)	84 (48.6%)	<0.001
Two yoghurts and/or 40 g cheese daily	1193 (43.84%)	239 (49.3%)	387 (41.1%)	343 (44%)	155 (45.2%)	68 (39.3%)	0.051
Sweets and candy several times a day	751 (27.6%)	134 (27.6%)	244 (25.9%)	226 (29%)	89 (25.9%)	58 (33.5%)	0.439

^1^ Results of the KIDMED test as a categorical variable. ^2^ *n* (%) indicate the number of participants who answered affirmatively to each item. Statistically significant results are in bold.

**Table 3:** Results of the multinomial logistic regression with categories of MD adherence as a dependent variable.

Predictors	Average MD Adherence		Good MD Adherence	
	OR (95% CI) ^1^	*p*-Value	OR (95% CI) ^1^	*p*-Value
Age	0.962 (0.933, 0.991)	0.011	0.974 (0.939, 1.011)	0.165
Number of daily meals on working days	1.242 (0.913, 1.688)	0.167	1.341 (0.919, 1.957)	0.128
Number of daily meals on day-offs	1.264 (0.910, 1.757)	0.162	1.339 (0.903, 1.986)	0.146
Number of snacks on working days	1.470 (1.157, 1.867)	0.002	1.978 (1.470, 2.660)	<0.001
Number of snacks on day-offs	0.773 (0.627, 0.953)	0.016	0.738 (0.567, 0.960)	0.023
Do you/Does your child participate in organized physical activity?				
No	1.016 (0.776, 1.332)	0.907	0.819 (0.589, 1.139)	0.235
**Predictors**	**Average MD Adherence**		**Good MD Adherence**	
	**OR (95% CI) ^1^**	***p*-Value**	**OR (95% CI) ^1^**	***p*-Value**
Yes	-	-	-	-
How many times a week do you (or does your child) do some sport, dance or play some game in which you are (or your child is) very active?				
None	0.365 (0.216, 0.615)	<0.001	0.283 (0.145, 0.550)	<0.001
1 time	0.416 (0.236, 0.734)	0.002	0.374 (0.182, 0.770)	0.008
2–3 times	0.676 (0.463, 0.987)	0.043	0.508 (0.330, 0.782)	0.002
4–5 times	1.222 (0.814, 1.834)	0.334	0.759 (0.478, 1.206)	0.243
6 or more times	-	-	-	-
Having breakfast together as a family				
No	0.644 (0.514, 0.808)	<0.001	0.311 (0.233, 0.416)	<0.001
Yes	-	-	-	-
Having dinner together as a family				
No	0.980 (0.698, 1.375)	0.906	0.789 (0.493, 1.261)	0.322
Yes	-	-	-	-

^1^ Odds ratios (OR) were calculated by multivariate multinomial logistic regression with low MD adherence as the reference category in the dependent variable.

**Table 4:** Results of the multinomial logistic regression where participant’s general health (self- or parent-perceived) was dependent variable while MD adherence and level of physical activity were independent variables.

Predictors	Good		Very Good	
	OR (95% CI)	*p*-Value	OR (95% CI)	*p*-Value
How many times a week do you (or does your child) do some sport, dance, or play some game in which you are (or your child is) very active?				
**Predictors**	**Good**		**Very Good**	
	**OR (95% CI)**	***p*-Value**	**OR (95% CI)**	***p*-Value**
None	10.335 (3.140, 34.021)	<0.001	7.353 (2.582, 20.940)	<0.001
1 time	4.626 (2.565, 8.343)	<0.001	4.259 (2.759, 6.573)	<0.001
2–3 times	1.626 (0.978, 2.705)	0.061	2.804 (1.990, 3.950)	<0.001
4–5 times	0.886 (0.515, 1.525)	0.662	2.196 (1.552, 3.108)	<0.001
6 or more times	-	-	-	-
Mediterranean index score classification				
Average MD adherence	0.596 (0.419, 0.848)	0.004	0.812 (0.643, 1.026)	0.081
Good MD adherence	0.497 (0.311, 0.794)	0.003	0.646 (0.485, 0.861)	0.003
Poor MD adherence	-	-	-	-

Odds ratios (OR) were calculated by multivariate multinomial logistic regression with excellent general health (self- or parent-perceived) as the reference category in the dependent variable. Participants’ general health was rated as excellent, very good, good, or fair/poor; however, no “fair/poor” rating was recorded in our sample.

## Data Availability

Raw data can be found at corresponding author via e-mail: antonela.matana@gmail.com.
